# Host Cell Invasion by Apicomplexan Parasites: The Junction Conundrum

**DOI:** 10.1371/journal.ppat.1004273

**Published:** 2014-09-18

**Authors:** Daniel Bargieri, Vanessa Lagal, Nicole Andenmatten, Isabelle Tardieux, Markus Meissner, Robert Ménard

**Affiliations:** 1 Institut Pasteur, Malaria Biology and Genetics Unit, Department of Parasitology and Mycology, Paris, France; 2 Institut Cochin, Laboratory Barriers and Pathogens, INSERM U-1016, CNRS UMR-8104, University of Paris Descartes, Paris, France; 3 Institute of Infection, Immunity and Inflammation, Wellcome Centre for Molecular Parasitology, Glasgow Biomedical Research Centre, University of Glasgow, Glasgow, United Kingdom; International Centre for Genetic Engineering and Biotechnology, India

## Introduction

Apicomplexans form a large phylum of parasitic protists, some of which cause severe diseases in humans. Most notorious is *Plasmodium*, the agent of malaria, which kills around a million people each year, mostly young children in Africa. Most successful is *Toxoplasma*, which parasitizes nearly a third of the human population, making those people at risk of life-threatening complications, primarily encephalitis or pneumonia, in case of immunosuppression. Other apicomplexans of human importance include *Cryptosporidium*, *Isospora,* and *Sarcocystis*, which are opportunistic pathogens that cause severe diarrhea often associated with AIDS. Several apicomplexan parasites cause heavy losses in livestock, particularly *Theileria* and *Babesia* in cattle and *Eimeria* in poultry.

Most apicomplexans are obligate intracellular parasites. Their extracellular stages, called zoites, display several conserved features: they are elongated and polarized cells, their shape is maintained by a set of microtubules running longitudinally, and their anterior pole contains secretory vesicles, called micronemes and rhoptries, which secrete their content at the anterior tip of the parasite. Most zoites also share two unique properties among eukaryotic cells. They move on substrate by a gliding type of motility, i.e., without overt deformation of the cell shape, at speeds of several microns per second. They also typically invade host cells by forming a ring-like junction with the host cell membrane. Zoites slide through the junction into an invagination of the host cell surface that becomes the parasitophorous vacuole (PV) after pinching off from the host cell plasma membrane, in a process that takes less than a minute. Once inside the PV niche, the zoite can multiply into multiple new zoites that eventually egress the infected cell to infect new host cells.

Much work has been performed since the late 1970s to understand the cellular and molecular bases of host cell invasion by apicomplexans, using various zoites as models. The overall invasion process encompasses several steps, including loose followed by intimate attachment, reorientation relative to the host cell surface, and organelle discharge with junction formation. The ultimate step, sliding through the junction inside the PV and called here internalization, is commonly viewed as powered by the zoite submembrane actin-myosin motor. The junction is thought to act as a traction point for the motor, to bridge the cortical cytoskeletons in the two cells, and to be made of parasite proteins conserved in the apicomplexan phylum. In this review, we confront these established notions with genetic data recently obtained in *Plasmodium* and *Toxoplasma* parasites.

### The Junction: From “Moving” to Stationary

The first observation of a junction between an apicomplexan zoite and its host cell was made using *Plasmodium* merozoites and their target cells, erythrocytes [1]. Electron microscopy showed that the merozoite, after initial random binding, reorients so that its apical tip faces the erythrocyte surface, and then induces a circumferential zone of close apposition of the zoite and erythrocyte membranes over ∼250 nm and the thickening of the inner leaflet of the erythrocyte membrane [1]. This junctional area was described as “actively moving down the body of the merozoite,” since the poorly motile merozoite was not thought to be capable of actively moving inside the cell, and was thus termed “moving junction” [1].

Studies in the 1980s focused on the highly motile *Eimeria* sporozoites. They showed that several activities at the zoite surface were dependent on parasite actin, including the posterior translocation (capping) of various surface ligands and beads [2]. Videomicroscopic studies revealed that host cell invasion by *Eimeria* sporozoites was continuous with extracellular gliding [3]. This led to the proposal that the zoite actin-based system would power both gliding motility and host cell invasion by capping substrate-binding ligands or the junction, respectively, which implied that the zoite actively moved inside the host cell [3], [4]. After myosins were identified in *Toxoplasma* [5] and in *Plasmodium* [6], it was assumed that an actin-myosin motor powered the zoite motile processes.

The role of the host cell during zoite invasion has been studied mainly with *Toxoplasma* tachyzoites, which can be made to invade host cells at high frequency and synchronicity. The host cell was initially described as displaying no detectable actin reorganization and playing no active role during tachyzoite invasion [7], [8]. More recent work found that *Toxoplasma* tachyzoites induced, specifically at the junction, host actin polymerization and recruitment of the Arp2/3 complex, an actin-nucleating factor, which is important for tachyzoite entry [9]. Videomicroscopic studies showed a stationary ring of host F-actin at the parasite constriction, in agreement with the junction acting as an anchor for zoite traction inside the cell. In addition to de novo actin polymerization at the junction, tachyzoite invasion also requires disorganization of the host cortical actin meshwork. This activity is in part dependent on Toxofilin [10], a *Toxoplasma* protein that sequesters actin monomers in vitro [11] and promotes actin turnover at the leading edge of the cell [10]. Localized actin disassembly might thus release G-actin necessary to feed actin reassembly at the junction, regulated by recruited Arp2/3 complex, to anchor the junction to the host cortical cytoskeleton.

### The Parasite Motor

**Figure 1:**
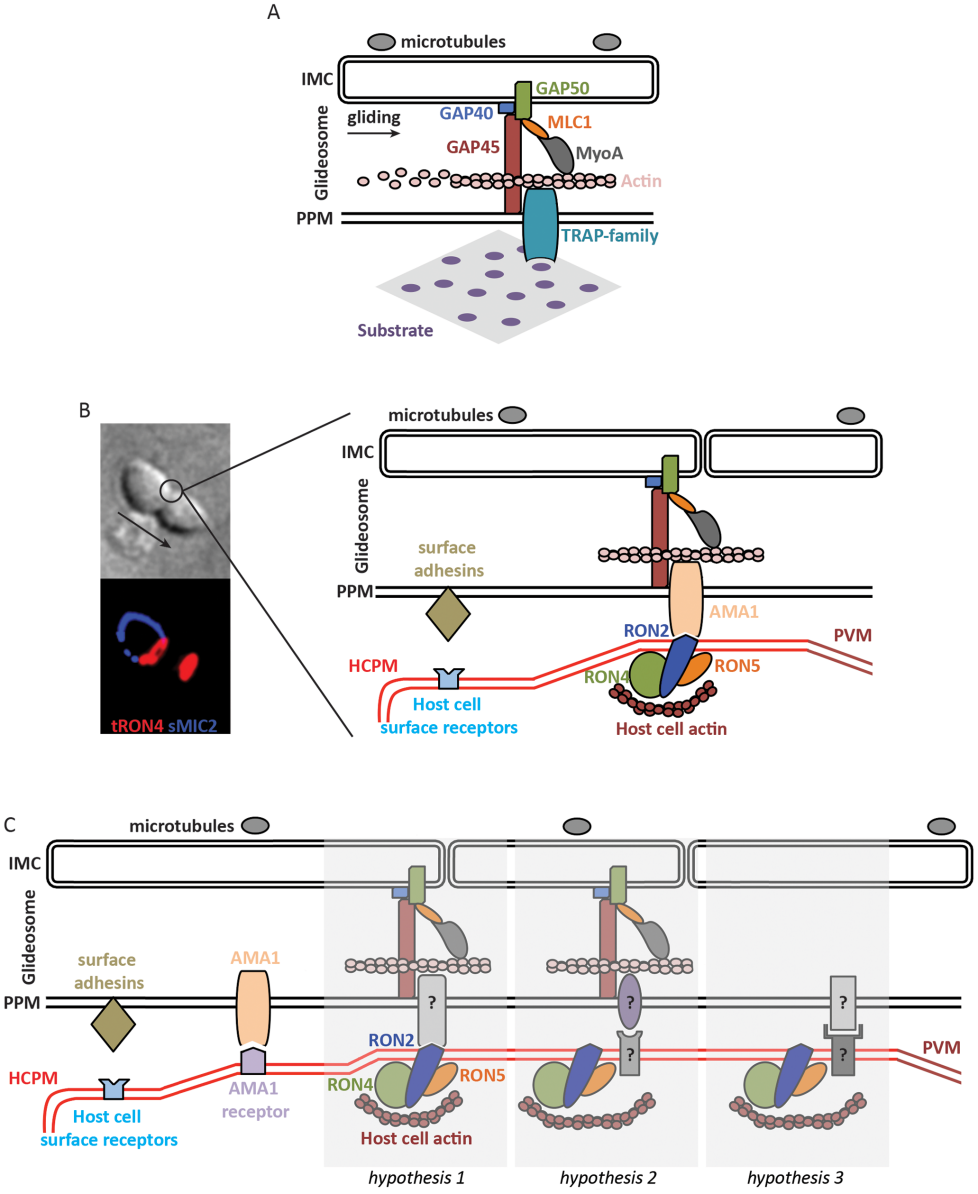
Molecular models of apicomplexan gliding and invasion. **A.** The parasite motor (glideosome) is located in the space between the parasite plasma membrane (PPM) and the inner membrane complex (IMC) apposed to the microtubules. Gliding motility is mediated by the binding of the ectodomain of transmembrane TRAP-family proteins to a solid substrate, while the cytoplasmic tail of the protein is linked to the parasite motor. The integrity of the glideosome is maintained by the gliding-associated protein 45 (GAP45), which is anchored to the PPM at one end and to the IMC, via GAPs 40 and 50, at the other end. The link between the GAPs, and ultimately the IMC, to actin is provided by Myosin A (MyoA) and the MyoA Light Chain 1 (MLC1). The movement of the cell is the consequence of the capping, by myosin-actin activity, of the TRAP-family protein. **B.** The model of invasion seen as the junction structured by the AMA1-RON complex. The figure on the left shows a *Toxoplasma* tachyzoite invading a host cell. The arrow indicates the direction of movement. Immunostaining of surface MIC2 (sMIC2) stains the part of the zoite cell still extracellular (blue), while the rest of the cell, already internalized, is not stained. Immunostaining of total RON4 (tRON4, red) marks the junction as a ring at the point of constriction, indicated by the circle, and the rhoptries at the apical pole of the zoite cell. After a first step of adhesion to the host cell plasma membrane (HCPM) mediated by parasite surface adhesins and host cell surface receptors, the binding of the transmembrane protein AMA1 to RON2, inserted at the host cell membrane and complexed with RONs 4 and 5, forms the junction. The link to the parasite motor is as in (A), while host actin recruited at the junction provides the link to the host cell cytoskeleton. The movement of the zoite towards the interior of the newly formed parasitophorous vacuole membrane (PVM) is thus a consequence of the capping of AMA1, which would be anchored at the junction by binding to RON2. **C.** Models of zoite invasion in which the functions of AMA1 and RONs are dissociated. Color codes and acronyms are as in (A) and (B). After a first step of adhesion mediated by parasite surface adhesins and host cell surface receptors, AMA1 binding to a host cell receptor provides a strong attachment between the zoite and host cell membranes, possibly leading to reorientation of the zoite to allow junction formation. Three different hypotheses could then explain junction formation: 1. A still-unknown transmembrane parasite protein binds to the motor and to RON2, taking the place previously assigned to AMA1. 2. Unknown proteins structure the junction and connect the parasite motor to the host cell cortical actin, in which case the role of the RONs at the junction is not structural. 3. Unknown proteins structure the junction without a role of the parasite motor during invasion.

The motor is located in the space (∼20 nm) between the zoite plasma membrane and the inner membrane complex (IMC), a continuous layer of flattened vesicles apposed onto the microtubule structure typical of alveolates (Figure 1A) [12]. The development in the 1990s of gene targeting techniques in *Toxoplasma* and *Plasmodium* has allowed the identification of some of the main players of the apicomplexan motor. The first proposed motor-substrate link was the thrombospondin-related anonymous protein (TRAP), a transmembrane protein of *Plasmodium* sporozoites conserved in the phylum that was found to be essential for sporozoite gliding [13]. The first (and, to date, only) myosin shown to operate during apicomplexan gliding is MyoA, a single-headed unconventional myosin of the apicomplexan-specific XIV class, which was shown to be crucial for gliding of *Toxoplasma* tachyzoites [14]. The orientation of the motor was determined by two main findings: the association of the cytoplasmic tails of *Plasmodium* TRAP and its MIC2 ortholog in *Toxoplasma* with actin [15] and the localization of the MyoA light chain (also called myosin A tail domain interacting protein, MTIP) in the IMC [16].

Actin polymerization in apicomplexans has been studied in some detail. In these parasites, which contain a limited repertoire of actin-binding factors [17], actin forms inherently short and unstable filaments [18], and normal zoite gliding requires rapid actin dynamics [19]. In the absence of Arp2/3 complex in these parasites, F-actin nucleation is promoted by formins [20], [21], which were detected as an apical ring at the junction, like actin itself [22], in the invading *Plasmodium* merozoite [20]. Actin depolymerization is controlled by actin-depolymerizing factor (ADF) [23] while profilin sequesters monomeric actin [24], [25]. How actin filaments are connected to the cytoplasmic tails of the TRAP/MIC2 proteins remains unclear. The link was originally thought to occur via aldolase [15], a glycolytic enzyme that binds the cytoplasmic tails of the TRAP/MIC2 proteins and is known to bundle actin filaments in mammalian cells. However, genetic data have now clearly shown that aldolase does not play such a mechanical, bridging role during gliding motility and invasion [26], [27] but is important for providing energy via its glycolytic activity [28]. Finally, structural components of the motor complex have also been identified, primarily in *Toxoplasma*, called gliding-associated protein 45 (GAP45) [29], [30], GAP50 [29], and GAP40 [31]. Although the individual contributions of the GAP proteins during gliding motility and invasion remain uncertain, they are thought to maintain the cohesion and integrity of the pellicle during zoite gliding and invasion, especially via GAP45 that spans the entire space between the plasma membrane and the IMC and anchors the motor complex at the IMC (Figure 1A) [31], [32].

The motor is still typically viewed as linear, i.e., as “linear arrangements of transmembrane proteins transducing the force generated by the actin-myosin motor and posteriorly capped,” as originally proposed by King [2]. However, recent studies using biophysical approaches to measure the force *Plasmodium* sporozoites exert on the surface during gliding indicate that zoite movement is not continuous, as predicted by the linear motor, but follows a stick-and-slip pattern [33]. This involves, in addition to backward capping of adhesion proteins, the formation/disengagement of adhesion sites at the front and rear ends of the zoite. Interestingly, TRAP appears to have a key role in the release of adhesion sites, not in retrograde capping (in stick but not in slip), while actin is important for both processes. Moreover, sporozoites lacking the motor-binding TRAP-like protein (TLP), which glide less efficiently by more frequently detaching from the substrate, were complemented by the addition of actin stabilizing drugs [34]. These data illustrate the complex bases of apicomplexan gliding, which may be more akin to crawling of mammalian cells than previously anticipated.

### What Is in the Junction?

#### Zoite-specific proteins?

Numerous proteins in *Plasmodium* merozoites have been described as being released from apical organelles, important for invasion and found at the junction. This is the case of several members of the micronemal erythrocyte binding like (EBL) family of proteins, such as *Plasmodium falciparum* erythrocyte-binding antigen 175 (EBA175) [35] and *P. knowlesi* Duffy binding protein (DBP) [36]. This is also the case of several rhoptry proteins, including the *P. falciparum* reticulocyte-binding like (RBL) homologue PfRh1 [37], PfRh2a [38], and PfRh5 [39], and of the rhoptry-associated leucine zipper-like protein 1 (RALP1) [40]. However, function is difficult to assess in the *Plasmodium* merozoite, typically relying on antibody inhibition and negative transfection experiments. In *Toxoplasma*, one member of the micronemal MIC family of proteins, MIC8, was shown to be specifically crucial for junction formation [41]. Importantly, none of these proteins is conserved across the phylum and most are stage-specific. If these proteins are indeed part of the junction, the latter might then be at least in part zoite-specific. If instead the junction is composed of a molecular core conserved across apicomplexans, then these stage-specific proteins may constitute adaptations to particular zoite-host cell combinations.

#### Motor-binding proteins?

Since the junction is viewed as a traction point for the motor, other candidates for junction components were the transmembrane proteins involved in gliding motility, i.e., the TRAP family of proteins including TRAP, TLP, and TRAP-related protein (TREP) in the *Plasmodium* sporozoite and MIC2 in the *Toxoplasma* tachyzoite [42]. However, there is no evidence that any of these proteins participates specifically at the junction during host cell invasion. In the *Toxoplasma* tachyzoite, inactivation of MIC2 impairs but does not preclude motility or invasion [43] and MIC2 is not specifically enriched at the junction during tachyzoite internalization. The hypothesis that TRAP/MIC2 might play a role as junction components was also favored by the presence in their extracellular domains of one or more A domains of von Willebrand factor, which are homologous to integrin I domains and thus potential ligands of host cell surface receptors [44]. However, the A domains of the TRAP family member CS and TRAP-related protein (CTRP), expressed by the *Plasmodium* motile ookinete stage that does not invade host cells, were shown to be important for gliding motility [45]. Together, these data favor the view that the TRAP family of proteins is involved in gliding motility, but not specifically for host cell entry.

#### AMA1-RON complexes?

In 2005, two papers identified a set of parasite proteins in *Toxoplasma* tachyzoites, called rhoptry neck proteins (RON), which specifically marked the constriction around invading zoites in a ring-like manner (Figure 1B) [46], [47]. Later, a ring-like staining of RON proteins was also shown in invading *Plasmodium* merozoites [48]. It was additionally found that in tachyzoite extracts, several RON proteins formed a complex with apical membrane antigen 1 (AMA1) [46], a transmembrane protein first identified in *Plasmodium* merozoites [49] and a leading malaria vaccine candidate. In humans, evidence suggests that AMA1 is an important target of naturally acquired protective antibodies preventing merozoite invasion of erythrocytes [50]. AMA1 vaccines have demonstrated protective efficacy in rodent and simian models against blood-stage challenge with the homologous strain [51], although human vaccine trials using AMA1 have shown poor efficacy so far [52].

Both AMA1 and RON proteins are conserved in the apicomplexan phylum and the AMA1-RON complex has now been detected in extracts from *Toxoplasma* tachyzoites and sporozoites [53], *Plasmodium* merozoites of various species [54], and *Neospora* [55]. Several independent lines of evidence have first favored the view that the AMA1-RON complex might constitute the building block of the junction. (i) The ectodomain of AMA1 binds RON2 [56], [57], which inserts into the host cell membrane [58] and is thought to bind to the cell cortical cytoskeleton via other RON proteins, a view recently strengthened by the observation that *Toxoplasma* RON4 may bind host cell tubulin [59]. (ii) The crystal structure of the AMA1–RON2 interaction in *Toxoplasma* [60] and *Plasmodium* [61] reveals a conserved RON2 loop that inserts deep into a hydrophobic groove in AMA1, suggesting that it might withstand mechanical forces and act as the traction point for the zoite motor. (iii) Antibodies or peptides that inhibit the AMA1–RON2 interaction reduce host cell invasion by *Toxoplasma* tachyzoites and *Plasmodium* merozoites [62]–[65]. These results clearly pointed to the view that cross-membrane AMA1-RON2 complexes shaped the junction for zoite internalization [66], [67] (Figure 1B). Consequently, the conserved AMA1–RON2 interaction has sparked much interest as a broad target for intervention against apicomplexan parasites [68], [69].

### AMA1-Dependent Attachment and AMA1-Independent Internalization

The development of conditional mutagenesis techniques in *Toxoplasma* [14], [70] and *Plasmodium* [71] has allowed the addressing of the functions of AMA1 and the RON complex (Table 1). All attempts to directly inactivate either *RON4* or *RON2* in *Toxoplasma* and *Plasmodium* have failed so far. *P. berghei RON4* conditional sporozoites, obtained by Flp/FRT-mediated recombination, are unable to invade hepatocytes [72]. *P. berghei RON2* conditional sporozoites, obtained by a promoter swap strategy, are unable to invade mosquito salivary gland cells [73]. *Toxoplasma gondii RON5* and *RON2* conditional tachyzoites, generated with a Tet-repressible promoter, are drastically impaired in invasion [74], [75]. Therefore, the RON proteins appear to play crucial roles during host cell invasion by all zoites.

**Table 1:** Mutants of interest in studies on host cell invasion by apicomplexans.

Gene	zoite	System	Phenotype	Ref.
RON4	*P. berghei* sporozoites	Flp/*FRT*	• Knock-down (KD) sporozoites do not invade hepatic cells in vitro	[72]
RON2	*P. berghei* sporozoites	Promoter swap	• RON2-negative sporozoites do not invade the mosquito salivary glands	[73]
RON5	*T. gondii* tachyzoites	Tet repression	• KD tachyzoites are unable to invade host cells	[74]
			• Loss of RON5 results in complete degradation of RON2 and mistargeting of RON4	
RON2	*T. gondii* tachyzoites	Tet repression	• KD tachyzoites display a severe block in host cell invasion	[75]
			• RON4 and RON5 are not properly localized within parasites	
AMA1	*T. gondii* tachyzoites	Tet repression	• KD tachyzoites do not progress from initial to intimate binding with the host cell membrane.	[78]
			• Rhoptry secretion is impaired and invasion is reduced to ∼15% that of WT.	
AMA1	*P. berghei* sporozoites and hepatic merozoites	Flp/*FRT*	• KD sporozoites normally invade hepatic cells in vitro and in vivo• KD hepatic merozoites cannot induce a blood infection in vivo	[72]
AMA1	*T. gondii* tachyzoites	Tet repression	• KD tachyzoites bind to host cells differently from WT	[72]
			• Internalization appears normal	
AMA1	*P. berghei* merozoites and sporozoites	Flp/*FRT* and direct knock-out	• Knock-out (KO) sporozoites normally invade hepatic cells in vitro• KO and KD merozoites are impaired in binding to erythrocytes. The growth rate of KO blood stages in vivo is ∼35% that of WT	[76]
AMA1	*T. gondii* tachyzoites	diCre/*loxP*	• KO tachyzoites bind to host cells with a distinct positioning relative to the host cell	[76]
			• Internalization appears normal but with decreased frequency (30%–40% that of WT)	
AMA1	*P. falciparum* merozoites	diCre/*loxP*	• Populations with 80% excised merozoites (with residual AMA1 due to late excision) show 37% reduction in invasion capacity	[77]
AMA1	*T. gondii* tachyzoites	Direct knock-out	• KO tachyzoites display ‘abortive invasions’	[75]
			• Residual invasion of AMA1KO tachyzoites is due to compensation by AMA1 paralogs	
MIC2	*T. gondii* tachyzoites	Tet repression	• KD tachyzoites are impaired in gliding. Attachment to host cells is reduced to 18% that of the parental strain	[43]
			• Invasion is reduced to 22% that of the parental strain.	
MIC2	*T. gondii* tachyzoites	diCre/*loxP*	• KO tachyzoites are clonally viable	[70]
			• Gliding motility and growth in cell monolayer are impaired	
TRAP	*P. berghei* sporozoites	Direct knock-out	• KO sporozoites are impaired in gliding motility. Invasion of mosquito salivary glands is impaired	[13]
			• Infection of mouse liver is compromised	
TRAP	*P. berghei* sporozoites	Direct knock-out	• KO sporozoites glide for one body length in both directions, while remaining attached to the substrate by one adhesion site	[33]
			• The turnover of adhesion sites is impaired. KO parasites cannot detach once attached	
TRAP tail	*P. berghei* sporozoites	Subtle mutagenesis	• Deletion of the entire cytoplasmic tail of TRAP renders sporozoites non-motile• Deletion of the distal third of the TRAP cytoplasmic tail causes a pendulum gliding	[100]
TREP	*P. berghei* sporozoites	Direct knock-out	• KO sporozoites are impaired in gliding motility	[71]
			• Invasion of mosquito salivary glands is impaired	
Aldolase	*T. gondii* tachyzoites	Tet repression	• KD tachyzoites are impaired in gliding motility and invasion	[27]
			• KD complemented with MIC2-binding impaired versions of aldolase display normal gliding	
Aldolase	*T. gondii* tachyzoites	diCre/*loxP*	• KO tachyzoites can be cloned and propagate when grown *in vitro* in glucose-free medium	[26]
Actin	*T. gondii* tachyzoites	diCre/*loxP*	• Gliding and invasion are 10% that of WT	[32]
			• KO tachyzoites invade through a junction and multiply, but are not clonally viable due to abnormal segregation of apicoplasts	
MyoA	*T. gondii* tachyzoites	Tet repression	• KD tachyzoites are impaired in gliding motility	[14]
			• Host cell invasion is reduced to ∼20% that of WT	
MyoA	*T. gondii* tachyzoites	diCre/*loxP*	• KO tachyzoites are clonally viable. Gliding and egress are impaired	[32]
			• Invasion is reduced to 16% that of WT. Internalization is through a RON4-stained junction	
GAP45	*T. gondii* tachyzoites	Tet repression	• Other motor components redistribute to the cytosol and the glideosome “collapses”	[31]
			• KD tachyzoites are impaired in gliding and egress. Invasion efficiency is 25% that of the parental strain	
GAP45	*T. gondii* tachyzoites	diCre/*loxP*	• KO tachyzoites grow up to 14 days in culture. The IMC looses contact to the PM and MyoA and MLC1 become cytosolic	[32]
			• KO tachyzoites can glide. Egress is impaired. Internalization is through a • RON4-stained junction and is reduced to 6% that of WT	
MLC1	*T. gondii* tachyzoites	diCre/*loxP*	• KO tachyzoites can be grown up to 14 days in culture. MyoA is mislocalized	[32]
			• Gliding and egress are impaired. Invasion is reduced to 28% that of WT. Internalization is through a RON4-stained junction	

In contrast, all *AMA1* knock-down (AMA1^KD^) or knock-out (AMA1^KO^) zoites constructed are still invasive. AMA1^KD^ *P. berghei* sporozoites invade hepatocytes 3-fold better than the wild type (WT) in vitro and in vivo [72], and AMA1^KO^ *Toxoplasma* tachyzoites and *P. berghei* merozoites are internalized into host cells indistinguishably from the WT, i.e. systematically form normal RON rings and are internalized at normal speed [72], [76]. Nonetheless, AMA1^KO^ *Toxoplasma* tachyzoites and *P. berghei* merozoites display reduced invasion efficiency [72], [76], [77], along with a major impairment in host cell attachment [72], [76]. A recent report suggested that the adhesion defect of AMA1^KO^ tachyzoites might be secondary to a failure to form a normal junction leading to parasite detachment from the cell, based on immunofluorescence (IF) assays of rhoptry secretion [75]. However, what causes rhoptry secretion is still unknown and no direct evidence was provided that AMA1^KO^ tachyzoites formed an abnormal junction before detaching. AMA1^KO^ tachyzoites observed by real-time imaging did not display abortive invasions and their attachment defect (upright instead of flattened positioning relative to the cell surface) concerned the entire population, irrespective of invasion [72], [76]. In *Plasmodium*, AMA1^KO^ merozoites were 15-fold less adhesive to erythrocytes than controls after only 3 minutes incubation, which cannot be accounted for by failed invasions given that merozoite invasion efficiency is less than 5% [76]. A primary role of AMA1 in zoite binding to host cells is also in agreement with earlier work showing that AMA1 mediates attachment of *Toxoplasma* tachyzoites [78] and *Plasmodium* merozoites [79] to their respective host cells, and that *Plasmodium* AMA1 binds erythrocytes [80]–[82] and to the erythrocyte membrane receptor Kx [83].

Recent work revealed that the *Toxoplasma* genome encodes paralogs of AMA1 and RON2 in specific combinations. In the tachyzoite, RON2 interacts with AMA1 or AMA2 and RON2_L1_ with AMA4 [75], while in the sporozoite RON2_L2_ interacts with AMA3 in a manner mutually exclusive with tachyzoite paralogs [84]. Therefore, the hypothesis was raised that AMA1 paralogs might account for the residual invasive capacity of AMA1^KO^ tachyzoites [75], [84]. In agreement with this, the latter were found, during parasite selection, to up-regulate AMA2 as well as the RON2_L1_–AMA4 pair [75]. However, evidence that these paralogs act at and/or structure the junction is lacking, and the weaker affinity of RON2 for AMA2 compared with AMA1 [75] is at odds with the fully efficient internalization of AMA1^KO^ [76] (supposedly mediated by AMA2). In *Plasmodium*, the compensation theory appears particularly unlikely. The AMA1^KD^ Flp/FRT sporozoites undergo *AMA1* excision after parasite selection and yet are 100% invasive [76]. Additionally, *Plasmodium* expresses no *AMA1 or RON2* paralog. The protein most closely related to AMA1 is the trans-membrane protein MAEBL, with which it shares the presence of a cysteine-rich domain but differs by an unrelated cytoplasmic tail and the absence of RON2-binding ability. MAEBL was shown by gene targeting in both *P. berghei* [85] and *P. falciparum* [86] sporozoites to function as a stage-specific adhesion; it mediates oocyst sporozoite binding to the mosquito salivary glands, but not internalization into hepatocytes. This further suggests that AMA1, its paralogs in *Toxoplasma*, and MAEBL in *Plasmodium* form a family of stage-specific, host cell–binding proteins.

### Current Hypotheses

If AMA1 primarily mediates zoite intimate binding to host cell surfaces, irrespective of RON2 interaction and junction assembly, what could be the role of the conserved and therefore important AMA1–RON2 interaction? AMA1 might still bind to RON2, possibly to help further stabilize the zoite prior to internalization, although direct evidence for such a step is still lacking. Alternatively, AMA1–RON2 interactions might serve to process AMA1 at the junction during internalization of the AMA1-covered zoite. For example, interaction with RON2 might serve to disengage the AMA1–host cell receptor interaction and help the zoite slide free inside the PV, separated from the vacuole membrane. In agreement with this, RON2 binding induces conformational changes in AMA1 [60], which might impact AMA1 processing by the substilisin-like protease SUB2 [87] or intramembrane rhomboids [88]. Likewise, RON2_L2_ binding alters AMA3, including allosterically in its membrane-proximal domain [84]. Such AMA processing function of the AMA–RON2 interactions would be dispensable for internalization and yet block invasion if perturbed, reconciling the inhibition and genetic data.

The contribution of the apparently essential RON proteins is also unclear. They might be structural components of the junction, by linking it to the host cell cytoskeleton (Figure 1C, hypothesis 1), or might not be part of the force-transducing link (Figure 1C, hypothesis 2). Perhaps favoring the latter, the RON proteins are present in apicomplexans that are not known to form a junction, like *Theileria* [89], which raises the possibility that another zoite–host cell interaction might structure the junction, possibly involving host cell receptor(s). In any hypothesis, the junction constitutes a traction point for zoite internalization into the host cell.

### Motor-Independent Entry

The *Toxoplasma* tachyzoite is ideal to study zoite invasion, not just due to the frequency of observable invasion events but also the genetic tractability of the parasite. The use of a transcriptional regulation system based on artificial Tet-transactivators (TATi) allowed the generation of knock-down mutants and the functional dissection of individual components of the motor (Table 1). As already said, knocking down *MIC2* [43] or *MyoA* [14] does not result in a complete block in host cell invasion. Even knocking down *GAP45*, while leading to the detachment of the IMC from the plasma membrane (PM) and the release of the motor complex in the cytosol, does not abolish host cell entry [31]. In contrast, as mentioned above, a knock-down for *MIC8* does not affect gliding motility but completely blocks host cell invasion due to an inability to form a junction [41]. These partial phenotypes were typically explained by the leakiness of the Tet-inducible system, but were also a hint that the motor might not be essential for host cell invasion.

The current adaptation of a conditional recombination system based on dimerizable Cre has allowed the construction of a series of tachyzoite mutants completely lacking individual components of the motor complex, including MyoA, GAP45, MLC1, and Act1 [32], [70]. All of these mutants are affected in invasion efficiency but retain some invasive capacity (Table 1). Strikingly, tachyzoites devoid of MyoA, MLC1, or Act1, which is a single copy gene in *Toxoplasma*, can invade host cells through a junction [32], demonstrating that at least junction formation is independent of connection to the motor. Moreover, GAP45^KO^ tachyzoites, in which the IMC detaches from the parasite membrane and MyoA and MLC1 become cytosolic, remain motile and also invade [32]. These genetic data suggest that tachyzoites can move and enter host cells without a functional motor. However, whether this motor-independent entry pathway is the normal pathway used by the WT, or an alternative pathway discernable only when the motor is not functional, remains to be seen.

How could tachyzoites with a deficient actin-myosin motor invade host cells? Until recently, actin polymerization and actin-myosin contraction were thought to underlie force generation during movement. However, this view is currently being challenged by new models, in which hydrodynamic forces generate changes in cell shape during motility [90]. Indeed, there is mounting evidence that osmotic pressure and hydrodynamic fluids are critical for motility of amoeboid cells, while the actin-myosin system is critical for the formation and release of attachment sites and associated traction forces [91]. A recent report demonstrates the poroelastic nature of the cytosol [92], where force can be generated by differences in hydrodynamic pressure that can be higher in one part of a cell than another, leading to tension. This pressure can be generated by actin-myosin activity or by the localized activation of osmogenic ion transporters in the plasma membrane [90]. In agreement with such a model, Na^+^/H^+^ antiporters have been implicated in invasion and egress of host cells by *Toxoplasma* tachyzoites [93]–[95], and monovalent ion concentrations have been involved in gliding motility and host cell invasion efficiencies in *Toxoplasma* [96], [97]. Based on this, a gelsolation model for gliding motility and zoite internalization, in which the acto-myosin system of the parasite is required as a clutch for force transmission but not for the generation of the force itself, has recently been proposed [32].

Another possibility that cannot be excluded is that the force for parasite internalization might originate from the host cell. *Theileria* sporozoites and merozoites, which lack an IMC and subpellicular microtubules and are not motile, invade host cells in any orientation, without a junction, and by a mechanism of circumferential zippering of parasite and host cell membranes [89]. Others, like *Cryptosporidium* sporozoites, are motile but rest on the host cell surface and induce the formation of host cell membrane folds that progressively encapsulate the “epicellular parasite” inside the PV [98]. Interestingly, these membrane protrusions recruit a host cell Na^+^/glucose cotransporter and aquaporin 1, which generate localized water influx and are required for parasite invasion [99].

If zoite internalization is powered by the host cell or by hydrodynamic forces, then the junction would no longer be connected to the parasite motor. In this case, the junction might serve as a membrane “seal,” possibly regulating protein processing upon entry into the PV, facilitating membrane dynamics, fluidity and curvature, and/or ensuring correct formation of the PV (Figure 1C, hypothesis 3).

## Conclusions

New mutagenesis data question the current view that apicomplexan zoites invade host cells by a unique pathway involving their motor and AMA1–RON2 interactions as traction points at the junction. AMA1 appears not to be involved in junction function and the RON proteins are the only parasite proteins known to functionally associate with the junction. Whether the RON complex transmits force at the junction remains uncertain, though the evidence of a link between the RON proteins and the host cell cytoskeleton points to this direction. The conservation of the RON proteins in apicomplexans and of their essential role in invasion suggests a conserved molecular kit for junction formation. Still, there is no definitive evidence for such a conserved apicomplexan junction core, and at least part of its structure might be stage-specific. Whether the zoite provides all pieces of the junction, or whether the host cell also provides receptors, possibly located in specific microdomains, is also unclear.

Moreover, it now appears that the force required for gliding motility and host cell entry might, at least in *Toxoplasma*, be generated in a motor-independent manner. Whether this holds true for other apicomplexan genera remains to be seen. In any event, apicomplexan invasion of host cells appears more complex than previously thought. Dissecting the process and its possible versatility will require establishing novel experimental approaches, including biophysical, and investigating unconventional force-generation means in zoites, as well as in host cells, that might facilitate, or even replace, the activity of the parasite motor.
